# The commonly used antibiotic streptomycin reduces protein synthesis and differentiation in cultured C2C12 myotubes

**DOI:** 10.14814/phy2.70353

**Published:** 2025-06-17

**Authors:** Chuqi He, Moritz Eggelbusch, Jelle Y. Huijts, Andi Shi, Gerard J. de Wit, Carla Offringa, Richard T. Jaspers, Rob C. I. Wüst

**Affiliations:** ^1^ Department of Human Movement Sciences, Faculty of Behavioural and Movement Sciences, Amsterdam Movement Sciences Vrije Universiteit Amsterdam Amsterdam The Netherlands; ^2^ Department of Nutrition and Dietetics Amsterdam University Medical Centers, Amsterdam Movement Sciences Amsterdam The Netherlands; ^3^ Center of Expertise Urban Vitality, Faculty of Sports and Nutrition Amsterdam University of Applied Sciences Amsterdam The Netherlands

**Keywords:** antibiotics, atrophy, metabolism, protein synthesis, skeletal muscle, streptomycin

## Abstract

The antibiotic streptomycin is an integral part of cell culture medium. Because streptomycin inhibits bacterial protein synthesis, streptomycin might also have off‐target effects on muscle cell function. Here, we studied the effect of streptomycin on C2C12 myoblasts, myofiber growth, and metabolism. C2C12 myoblasts were cultured with or without streptomycin. The control condition consisted of carbenicillin and ampicillin. Streptomycin did not impair myoblast proliferation rate. Streptomycin exposure led to a ~ 40% reduction in myotube diameter and reduced protein synthesis rate. Myotubes with streptomycin showed a 25% lower differentiation and 60% lower fusion index. Expression of cell stress markers was upregulated by streptomycin. Mitochondrial respiration rate was unaffected by streptomycin, but gene expression levels of *Myh3* and *Acta1* were lower, as well as the protein content of mitochondrial complex I subunits. Myotubes cultured in the presence of streptomycin showed fragmentation of the mitochondrial network, a smaller mitochondrial footprint (−64%), and shorter branch lengths (−34%). Streptomycin does not alter C2C12 myoblast proliferation but reduces global protein synthesis rates in differentiating myotubes. The routine use of streptomycin in muscle cell cultures should be carefully evaluated, particularly when investigating muscle growth, metabolism, or protein synthesis, where off‐target effects may confound experimental outcomes.

## 1 INTRODUCTION

Antibiotics are commonly prescribed drugs to treat infectious diseases, and are used during organ transplantation and invasive cardiac surgeries (Hutchings et al., 2019). Antibiotics are generally regarded as safe, and prescribed to 30% of outpatients annually (Shallcross et al., 2017) (Shapiro et al., 2014). Roughly 70% of hospitalized patients admitted to the intensive care unit (ICU) across 88 nations were given at least one antibiotic (Olesen et al., 2018). Over half of the critically ill patients diagnosed with SARS‐CoV‐2 infection were prescribed antibiotics, a figure that is considerably higher than the projected rate of diagnosed bacterial infections (Abu‐Rub et al., 2021; Rawson et al., 2020). Despite the common assumption of a high level of safety, some widely used antibiotics, such as doxycycline, metronidazole, azithromycin, and clarithromycin, are associated with the development of metabolic dysfunction (Kose & Wakabayashi, 2020; Manickam et al., 2018; Ray et al., 2012; Veronese et al., 2017; Wüst et al., 2020), which is related to an increase in cardiovascular mortality (Ray et al., 2012), and correlate with the long‐term risk of acute myocardial infarction, stroke, and mortality (Mosholder et al., 2018). Patients with chronic disease often experience skeletal muscle atrophy (Cao et al., 2018; Ding et al., 2018), accompanied by a reduction in maximal force and metabolic dysfunction (Mrowka & Westphal, 2018), overall predisposing affected individuals to increased all‐cause morbidity and mortality rates (García‐Hermoso et al., 2018; Nichols et al., 2019; Sipers et al., 2019). Whether the use of antibiotics affects skeletal muscle growth and metabolism is, however, currently unknown.

The most widespread antibiotics used for cell culture to prevent contamination with microbiological organisms are a combination of penicillin and streptomycin (Ryu et al., 2017). The class of penicillin affects bacterial cell wall integrity and is not known to have off‐target effects (Erlanger & Goode, 1967). Streptomycin is an aminoglycoside antibiotic and is used ubiquitously due to its effectiveness and low cost. Streptomycin exerts its function by inhibiting bacterial protein synthesis. Yet, bacteria are the ancestors of mitochondria, and as such, streptomycin may also act on mitochondria in eukaryotic cells (Archibald, 2015). The mitochondrial ribosome (mitoribosome) is considered the primary intracellular target of streptomycin in eukaryotic cells due to its circular mitochondrial DNA (Eustice & Wilhelm, 1984). Therefore, streptomycin may induce a disruption in mitochondrial protein synthesis and interference with mitochondrial function (Wüst et al., 2021). Accordingly, streptomycin has been shown to inhibit general protein synthesis in primary hepatocyte cultures (Schwarze & Seglen, 1981) and cultured brain cells of newborn mice (Amonn et al., 1978), and may also interfere with miRNAs (Relier et al., 2016). Meanwhile, some studies suggest that a cocktail of penicillin/streptomycin negatively affects cell differentiation in keratinocytes (Nygaard et al., 2015), human adipose tissue‐derived stem cells (Llobet et al., 2015), and embryonic stem cells (Cohen et al., 2006). These findings highlight mitochondrial dysfunction and reduced protein synthesis rate as potential off‐target effects of streptomycin (Serio et al., 2018).

Mitochondrial function and protein synthesis are crucial aspects of muscle cell function, particularly in tissue with high energy demands, such as skeletal muscle cells. If streptomycin alters gene expression profiles of muscle cells and impairs the rate of proliferation and differentiation, this could also complicate the interpretation of cell culture studies, as metabolic interventions can alternatively be interpreted using a second‐hit model (Wüst et al., 2020). The aim of this study was to assess whether streptomycin exposure affects myoblast proliferation and differentiation into myotubes. Myotube growth and mitochondrial function were assessed to study how streptomycin affects hypertrophic and metabolic pathways. We hypothesized that the use of streptomycin results in smaller myotube diameters and that mitochondrial protein synthesis and energy metabolism in C2C12 myotubes are negatively affected by streptomycin exposure. C2C12 myoblasts were chosen for this study due to their well‐characterized ability to differentiate into multinucleated myotubes, providing a reliable in vitro model for skeletal muscle development and function (Li et al., 2023). Differentiated C2C12 myotubes recapitulate key metabolic and contractile properties of skeletal muscle, including mitochondrial biogenesis and oxidative phosphorylation, making them particularly suitable for investigating the effects of streptomycin on muscle differentiation and mitochondrial function (Wong et al., 2020). We used ampicillin and carbenicillin as control antibiotics, which are broad‐spectrum antibiotics for treating gram‐positive and gram‐negative bacteria, respectively. They target the bacterial cell wall, so they are not likely to interfere with eukaryotic cell functions, thus acting as a control group.

## 2 MATERIALS AND METHODS

### 2.1 Cell culture

C2C12 mouse myoblast cells (ATCC, Manassas, VA, USA) were cultured in growth medium, consisting of high‐glucose Dulbecco's Modified Eagle Medium (DMEM, Gibco, USA; 11,995–065) with 10% fetal bovine serum (Biowest, Nuaillé, France; S181BH‐500), and 0.5% amphotericin B (Gibco, USA; 15,290–018). When cell densities reached 80–90% confluence, growth medium was replaced by differentiation medium which was refreshed daily for 6 days and consisted of high‐glucose DMEM supplemented with 2% horse serum (Biowest, Nuaillé, France; S0910) and 0.5% amphotericin B. Myotubes began forming within the 6‐day differentiation period.

We used the following antibiotics during the proliferation and differentiation phases at the concentrations recommended for cell culture experiments, according to the standard cell culture protocols listed by the American Type Culture Collection (ATCC), which is widely adopted in laboratories worldwide (American Type Culture Collection, 2014): 100 μg/mL carbenicillin (Sigma‐Aldrich, Amersham, USA; C1389) and 100 μg/mL ampicillin (Sigma‐Aldrich, Amersham, USA; A8351), or 100 IU/mL penicillin (Gibco, USA; 15140–122) and 100 μg/mL streptomycin (Fisher Scientific, USA; 455340250), or 100 μg/mL carbenicillin, 100 μg/mL ampicillin, and 100 μg/mL streptomycin. Antibiotic concentration was not lowered due to potential negative effects of bacterial growth on myotube growth and mitochondrial structure (Eggelbusch et al., 2022). Although they do not directly correspond to clinical antibiotic doses, they are relevant for in vitro models investigating the potential off‐target effects of antibiotics on mammalian cells. Cells were maintained at 37°C with a 5% CO_2_ atmosphere, and cell passages were maintained  below passage 15.

### 2.2 Cell proliferation

To assess the proliferation rate of C2C12 myoblasts, an EdU (5‐ethynyl‐2′‐deoxyuridine) assay was performed with a Click‐iT EdU kit (Fisher Scientific, USA; C10640). C2C12 myoblasts were seeded in 24‐well plates (Greiner bio‐one, Netherlands, 662,160) and incubated in growth medium with different antibiotic combinations (PS, CA, or CAS). After 24–48 hours, cells were incubated with 10 μM EdU reagent for 2 hours at 37°C and subsequently fixed with 3.7% formaldehyde in phosphate‐buffered saline (PBS) at room temperature for 15 min. Cells were permeabilized with 0.5% Triton‐X100 for 10 min, then stained with the Click‐iT reaction cocktail for 30 min in the dark. Next, cells were stained with DAPI (VECTASHIELD, Vector Laboratories, USA; H‐1200) to visualize myonuclei, and images were taken using a fluorescence microscope at 10x magnification (ZEISS Axiovert 200 M, Carl Zeiss Microscopy, Jena, Germany). The percentage of EdU positive cells (with emission 350 nm/excitation 461 nm) was calculated based on average of 5 images per biological replicate.

### 2.3 Differentiation and fusion index of myotubes

To analyze differentiation rates and myotube morphology, immunofluorescent staining for the muscle‐specific marker myosin heavy chain (MyHC) was performed on C2C12 myotubes, as described before (Shi et al., 2021). In short, C2C12 myoblasts were seeded in 24‐well plates (Greiner bio‐one, Netherlands, 662,160) and cultured in standard growth medium. Growth medium was removed when cells reached 80–90% confluence and replaced by differentiation medium with different antibiotics (CA, PS, or CAS). After 6 days, myotubes were fixed in 4% paraformaldehyde, permeabilized in 0.5% Triton X‐100, and blocked in 0.025% Tween‐20 in DPBS. Subsequently, cell were incubated in 5% normal goat serum (NGS, ThermoFisher Scientific, USA; 50062Z) at room temperature for 1 hour. Cells were incubated overnight at 4°C with mouse anti‐myosin monoclonal antibody (MF‐20, 1:50, Developmental Studies Hybridoma Bank, Iowa City, USA; AB2147781), followed by Alexa Fluor 488‐conjugated goat anti‐mouse IgG2b Cross‐Adsorbed secondary antibody (1:1000, Invitrogen, Carlsbad, CA, USA; A21141) for 1 hour and Hoechst staining. Images were taken using a fluorescence microscope (ZEISS Axiovert 200 M, Carl Zeiss Microscopy, Jena, Germany). Five images were taken per well, and 10–15 myotubes per image. Total nuclei number, number of nuclei within myotube, and myotube number were counted using ImageJ software (NIH, Bethesda, USA) to subsequently calculate the myotube differentiation index (differentiated nuclei/total nuclei) and fusion index (nuclei within fused myotubes /total nuclei).

### 2.4 Myotube diameter

Myotube size was assessed from myotube images after 6 days of differentiation, as described before (Eggelbusch et al., 2022). For this, 10 randomly selected fields per well were photographed with at least 200 myotubes analyzed per biological replicate. The average size of each myotube was calculated as the mean of 3 diametric measurements taken at equidistant intervals along its length (ImageJ, NIH, Bethesda, USA).

### 2.5 Rate of protein synthesis

To measure the general protein synthesis rate of C2C12 myotubes, a puromycin incorporation SUnSET assay was performed (Goodman & Hornberger, 2013). Myotubes were differentiated as described above. After 6 days of differentiation in the different cell culture media, 1 μM puromycin (Merck Millipore, San Diego, CA, USA; P8833) was added to all wells precisely 30 min before cells were lysed for western immunoblotting. Equal loading of was verified by normalizing to pan‐actin (rabbit polyclonal antibody, 1:4000, Cell Signaling Technology, USA; D18C11). Quantification was performed using ImageJ software.

### 2.6 Protein content by Western immunoblotting

Protein contents of key markers of protein synthesis, degradation, and cellular metabolism were assessed by Western immunoblotting, as described before (Eggelbusch et al., 2022). Total protein was obtained from each sample with a RIPA lysis buffer (Sigma‐Aldrich, Amersham, USA; R0278) containing protease inhibitor (Sigma‐Aldrich, Amersham, USA; 11,836,153,001) and phosphatase inhibitor (Sigma‐Aldrich, Amersham, USA; 04906837001). Cell lysates were centrifuged to pellet debris, and the total protein concentration of the supernatant was measured using the Pierce BCA Protein Assay Kit (Thermo Fisher Scientific, Waltham, MA, USA; 23,225). Samples were run on a gel using a 15% SDS‐PAGE, and proteins were transferred onto polyvinylidene difluoride membranes. Membranes were blocked to prevent nonspecific binding with 5% nonfat dry milk in PBS, followed by overnight incubation at 4°C with primary antibodies. The following primary antibodies were used: mouse anti‐puromycin (mouse monoclonal antibody, d1:1000, Merck Millipore, USA; 12D10); OXPHOS (rodent antibody cocktail, 1:1000, Thermo Fisher Scientific, USA, XC3517569); phospho‐p70S6K (rabbit monoclonal antibody, 1:1000, Cell Signaling Technology, USA; 49D7) and p70S6K (rabbit monoclonal antibody 1:1000, Cell Signaling Technology, USA; 108D2); pan‐actin (rabbit polyclonal antibody, 1:4000, Cell Signaling Technology, USA; D18C11); vinculin (rabbit polyclonal antibody, 1:4000, Cell Signaling Technology, USA; D43B3). After three 5‐min wash steps with 0.01% TBS‐T, membranes were incubated with a secondary antibody (polyclonal goat anti‐rabbit IgG, 1:2000, ThermoFisher Scientific, USA, A21428; polyclonal goat anti‐mouse IgG, 1:2000, ThermoFisher Scientific, USA, A11001) for 1 h at room temperature. Protein bands were detected using Western Blotting Detection Kit ECL Select (Sigma‐Aldrich, Amersham, USA; RPN2235). Images were taken with an Image Quant LAS‐500 (GE Healthcare, Bio‐Sciences). Blots were normalized to vinculin or pan‐actin as loading controls. Quantification was performed using ImageJ software.

### 2.7 Gene expression by RT‐qPCR

To study the initial effects of different antibiotics on skeletal muscle gene expression, real‐time qPCR (RT‐qPCR) was conducted, as previously described (Eggelbusch et al., 2022). C2C12 myotubes exposed to CA, PS, or CAS were harvested after six days of differentiation, homogenized in TRI Reagent (Invitrogen, Carlsbad, CA, USA; 11,312,940) and total RNA was isolated using the RiboPure™Kit (ThermoFisher Scientific, USA; AM1924). RNA concentration was quantitated using the NanoDrop 2000c spectrophotometer (Thermofisher Scientific). Then, RNA samples were converted to cDNA by reverse transcription using the SuperScript VILO MasterMix (Applied Biosystems, Foster City, CA, USA; 43,889,850) and amplified using the Power up SYBR Green Master Mix (ThermoFisher Scientific, USA; A25742) on the QuantStudio 3 Real‐Time PCR System (Applied Biosystems, Foster City, CA, USA). The following genes and primers were selected: myogenic differentiation 1 (*Myod*) for myotube differentiation; myosin, heavy polypeptide 3, skeletal muscle, embryonic (*Myh3*) and actin alpha 1, skeletal muscle (*Acta1*) as markers for the contractile machinery; insulin‐like growth factor 1 (*Igf1*) for protein synthesis; tripartite motif‐containing 63 (*Trim63*) and F‐box protein 32 (*Fbxo32*) to assess markers for protein degradation; eukaryotic translation initiation factor 2 alpha kinase 1 (*Hri*), eukaryotic translation initiation factor 2 alpha kinase 3 (*Perk*), and activating transcription factor 4 (*Atf4*) for cellular stress response; succinate dehydrogenase complex, subunit B, iron sulfur (*Sdhb*), cytochrome c oxidase II, mitochondrial (*mt‐Co2*), and peroxisome proliferative activated receptor, gamma, coactivator 1 alpha (*Ppargc1a*) for mitochondrial metabolism. *18S* was used as a housekeeping gene. All gene expression levels were normalized to *18S* using the delta CT method. Table 1 shows all primer sets used.

**TABLE 1:** Primer sequences used for q‐PCR.

Target gene	Forward sequence	Reverse sequence
*18S*	GTAACCCGTTGAACCCCATT	CCATCCAATCGGTAGTAGCG
*Igf1*	GTGTTGCTTCCGGAGCTGTG	CAAATGTACTTCCTTCTGAGTC
*Trim63*	GGGCTACCTTCCTCTCAAGTGC	CGTCCAGAGCGTGTCTCACTC
*Fbxo32*	AGACTGGACTTCTCGACTGC	TCAGCTCCAACAACAGCCTTACT
*Hri*	GGGCATAGCTCGGAATTGGA	TGGTACCGAACCTCCGTCT
*Perk*	TCGCGGCAGGTCCTTG	ACGTCCAAATCCCACTGCTT
*Atf4*	CCACCATGGCGTATTAGAGG	CAACACTGCTGCTGGATTTC
*Myod*	AGCACTACAGTGGCGACTCA	GCTCCACTATGCTGGACAGG
*Myh3*	CGCAGAATCGCAAGTCAATA	CAGGAGGTCTTGCTCACTCC
*Acta1*	GGCCAGAGTCAGAGCAGCAGAAAC	CACCAGGCCAGAGCCGTTGT
*Sdhb*	GTCAGGAGCCAAAATGGCG	CGACAGGCCTGAAACTGC
*mt‐Co2*	AACCGAGTCGTTCTGCCAAT	GGACTGCTCATGAGTGGAGG
*Ppargc1a*	ACACAACCGCAGTCGCAACA	GGGAACCCTTGGGGTCATTTGG

### 2.8 Mitochondrial morphology

To visualize mitochondria, cells were seeded on 8‐well ibidi plates (ibidi, Martinsried, Germany, 80,826) and cultured under the same conditions as described above. Cells were stained with MitoTracker™ Green FM (Thermofisher Scientific; USA; M7514) for 15 min, and mitochondrial networks were visualized by confocal microscopy (Nikon AXR, Tokyo, Japan). All images were acquired using identical laser power and exposure settings. At least 50 cells were analyzed per biological replicate. After acquisition, images were deconvoluted using Huygens Professional version 22.10 (Scientific Volume Imaging, Hilversum, The Netherlands). Quantification was performed with the Mitochondrial Network Analysis (MiNA) macro tool in ImageJ (Valente et al., 2017). Images were converted to a binary format. Afterwards, via skeletonization, images were converted into a wireframe composed of lines one pixel wide. This facilitated the computation of four parameters by the Mitochondrial Network Analysis (MiNA) macro tool to quantitatively capture the morphology of the mitochondrial network, namely mitochondrial footprint (the area or volume of the image consumed by mitochondrial signal), branch length mean (the mean length of all the lines used to represent the mitochondrial structures), summed branch lengths mean (the mean of the sum of the lengths of branches for each independent structure), and network branches mean (the mean number of attached lines used to represent each structure).

### 2.9 Mitochondrial respiration

Oxygen consumption and extracellular acidification rate were measured in myotubes using the Seahorse Extracellular Flux Analyzer (XFe96, Seahorse Bioscience, Agilent, USA), as described before (Gu et al., 2021; Wüst et al., 2021). After 6 days of differentiation, myotubes exposed to CA, PS, or CAS were washed twice and the medium was replaced with Seahorse Assay Medium: 10 mM glucose (Seahorse Bioscience, Agilent, USA; 103,577), 2 mM glutamine (Seahorse Bioscience, Agilent, USA;103,579) and 1 mM pyruvate (Seahorse Bioscience, Agilent, USA; 103,578), 5 mM HEPES (Seahorse Bioscience, Agilent, USA; 103,337), and 1.3 mM bicarbonate (Seahorse Bioscience, Agilent, USA; 103,015–100), pH 7.4. Then myotubes were incubated in a non‐CO_2_ incubator at 37°C for 45 mins. The Seahorse XF Cell Mito Stress Test Kit (Seahorse Bioscience, Agilent, USA; 103,015) was used: oxygen utilization and extracellular acidification rates were recorded for 1 hour, after which ATP synthase was blocked with 2 μM oligomycin, followed by an uncoupling of the inner‐mitochondrial membrane by FCCP (0.75 μM). Non‐mitochondrial respiration was measured after the addition of 0.5 μM rotenone and 0.5 μM antimycin A, and subtracted from all other values.

### 2.10 Statistical analysis

To compare results between groups, one‐way ANOVA with Bonferroni‐corrected post hoc analyses were performed. For post‐hoc analyses, we compared all results to PS, as the use of penicillin and streptomycin is common practice for cell culture, and PS was thus regarded as the ‘standard’. Statistical significance was defined as *p*‐values <0.05. Statistical analyses were performed with IBM SPSS Statistics version 23 (IBM, Amsterdam, The Netherlands). Results from 4 to 6 independent experiments are presented as mean ± standard deviation (SD).

## 3 RESULTS

### 3.1 Streptomycin does not affect C2C12 proliferation

**FIGURE 1:**
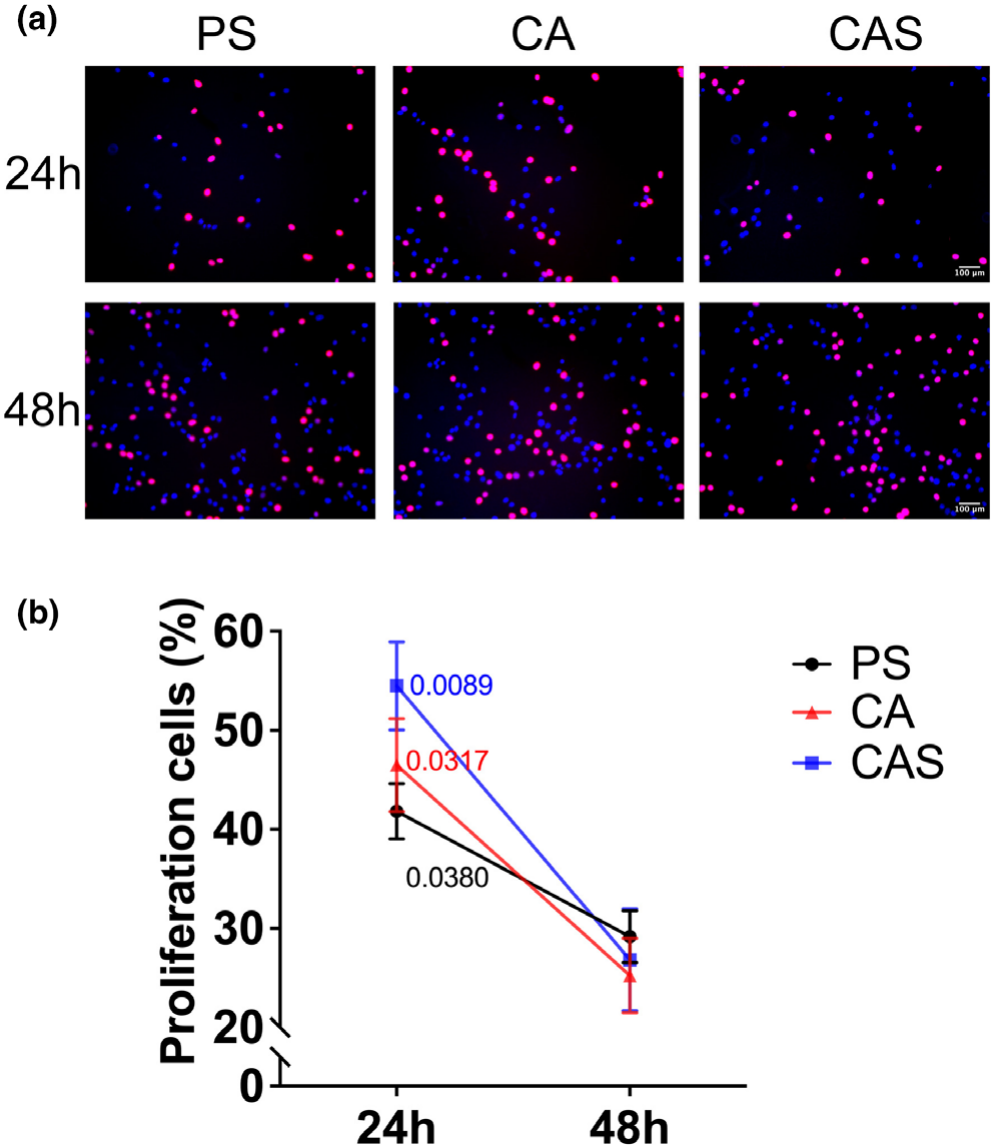
Streptomycin does not affect C2C12 cell proliferation. (a) Typical example images showing EdU‐positive (indicative of proliferating cells) nuclei stained in red, and nuclei were stained with Hoechst in blue. Scale bar: 100 μm. PS: Penicillin+streptomycin. CA: Carbenicillin+ampicillin. CAS: Carbenicillin + ampicillin + streptomycin (b) Percentage of EdU‐positive cells exposed to penicillin+streptomycin (PS), carbenicillin+ampicillin (CA) or carbenicillin+ampicillin+streptomycin (CAS) at 24 h and 48 h. Values are expressed as mean ± SD (*n* = 6).

We first assessed the effect of streptomycin on the proliferation rate of C2C12 myoblasts at 24 and 48 hours post‐exposure. Proliferation was not assessed beyond 48 hours, as cells begin to differentiate under our culture conditions at later time point, which could confound the interpretation. As shown in Figure 1, proliferation was significantly lower after 48 h compared to 24 h, but no differences between antibiotic conditions were observed at either time point, indicating that streptomycin does not affect the proliferation rate of C2C12 myoblasts.

### 3.2 Streptomycin inhibits C2C12 myotube differentiation and reduces myotube size

**FIGURE 2:**
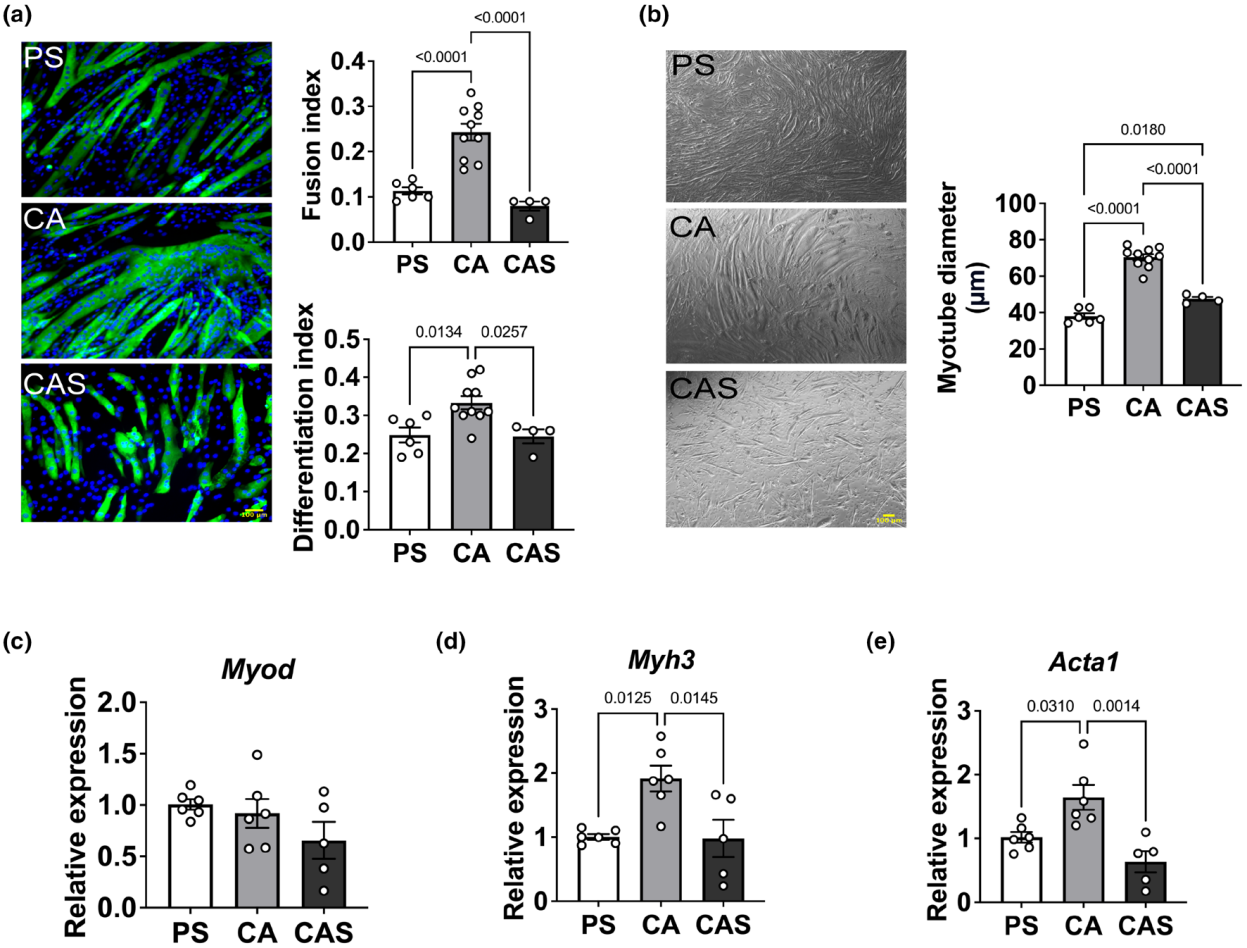
Streptomycin inhibits myotube differentiation and impairs myotube growth. (a) Representative results of immunofluorescence staining for myosin heavy chain (MyHC, green) and nuclei (blue) of myotubes in differentiation medium with different antibiotics on day 6. Myotube differentiation index (differentiated nuclei/total nuclei), and fusion index (fused tubes nuclei/total nuclei) of the cells exposed to penicillin+streptomycin (PS), carbenicillin+ampicillin (CA) or carbenicillin+ampicillin+streptomycin (CAS) for 6 days. Scale bar: 100 μm. Values are expressed as mean ± SD (*n* = 4–10). (b) Representative brightfield images and quantification of myotube diameter after 6 days of incubation in differentiation medium containing different antibiotics. Scale bar: 100 μm. Values are expressed as mean ± SD (*n* = 4–10). (c‐e) Genes related to contractility (*Myod*, *Myh3*, *Acta1*) after exposure to different differentiation media for 6 days. *Myod* was not different between groups (c). *Myh3* (d) and *Acta1* (e) were significantly lower in conditions where streptomycin was present. 18S rRNA was used as internal control for normalization. Values are expressed as mean ± SD (*n* = 5–6). PS: Penicillin+streptomycin. CA: Carbenicillin+ampicillin. CAS: Carbenicillin + ampicillin + streptomycin.

We next examined the effect of streptomycin on C2C12 differentiation. Figure 2a shows representative fluorescence images of myotubes exposed to different antibiotics. Whether streptomycin influences myotube formation was determined through quantitative analysis of myosin heavy chain (MHC) and nuclei staining. During differentiation, cells exposed to streptomycin showed significantly lower differentiation index (−25%) and fusion index (− ~ 60%), compared to cells exposed to carbenicillin and ampicillin (Figure 2a). Figure 2b shows brightfield images of myotubes after culture in the various media. Myotube diameter was significantly lower after penicillin/streptomycin (−46%) and carbenicillin/ampicillin/streptomycin (−32%) exposure, compared to carbenicillin/ampicillin only (Figure 2b). Next, we assessed expression levels of genes related to muscle contractility (*Myod, Myh3, Acta1*; Figure 2c–e). We observed significantly lower gene expression levels *of Myh3* (−48%) and *Acta1* (−50%) after exposure to streptomycin. Collectively, these findings indicate that streptomycin impaired C2C12 differentiation and myotube growth, potentially by suppressing the expression of key contractile genes.

### 3.3 Streptomycin reduces protein synthesis in C2C12 myotubes

**FIGURE 3:**
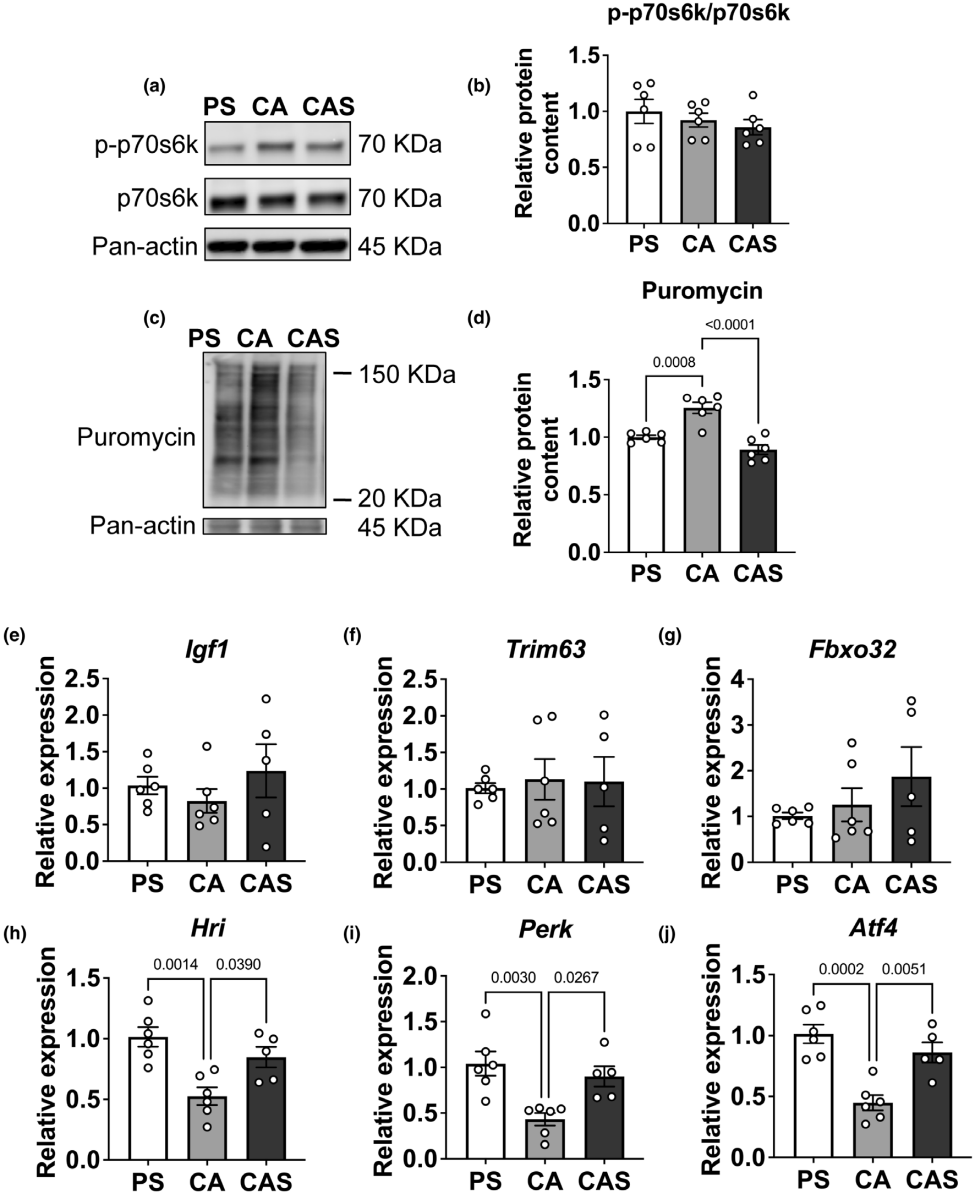
Streptomycin reduced protein synthesis rates in C2C12 myotubes. (a) Expression of p‐p70S6K and p70S6K in myotubes in differentiation medium with different antibiotics on day 6. Pan‐Actin was used as a loading control. (b) Changes in the p‐p70S6K/p70S6K ratio after different antibiotics exposure. (c) Western immunoblotting of puromycin, indicative of protein synthesis rates in myotubes. Pan‐Actin was used as a loading control. (d) Quantification of relative puromycin incorporation in myotubes after 6 days in differentiation medium with exposure to different antibiotics. Values are expressed as mean ± SD (*n* = 6). (e) Genes related to protein synthesis (*Igf1*) were not different between groups exposed to different differentiation mediums for 6 days. (f‐g) Genes related to protein degradation (*Trim63*, *Fbxo32*) were not different between groups. (h–j) Genes related to cell stress response (*Hri, Perk, Atf4*) was significantly higher in conditions where streptomycin was present. 18S rRNA was used as internal control for normalization. Values are expressed as mean ± SD (*n* = 5–6). PS: Penicillin+streptomycin. CA: Carbenicillin+ampicillin. CAS: Carbenicillin + ampicillin + streptomycin.

To confirm whether streptomycin affects protein synthesis rate, we first assessed the protein expression of p70S6K and phosphorylated p70S6K (p‐p70S6K) by Western immunoblotting (Figure 3a). The p‐p70S6k/p70S6K ratio did not differ among the three antibiotic conditions (Figure 3b). We next measured overall protein synthesis rates in C2C12 myotubes using a puromycin incorporation assay. Myotubes were exposed to puromycin for 30 minutes, and puromycin incorporation into proteins was quantified by Western blotting (Figure 3c). Puromycin signal intensity was significantly reduced (−25%) in myotubes exposed to streptomycin compared to those cultured with carbenicillin and ampicillin alone (Figure 3d). Expression levels of genes related to protein synthesis (*Igf1*) and protein degradation (*Trim63* and *Fbxo32*) were not different between groups (Figure 3e–g). However, the expression of stress response genes *Hri*, *Perk* and *Atf4* was significantly upregulated in streptomycin‐treated conditions, by 50%, 58% and 58%, respectively (Figure 3h–j). The decreased incorporation of puromycin in C2C12 myotubes indicates a reduction in protein synthesis upon streptomycin exposure, independent of alterations in certain protein and gene expression levels, but accompanied by the upregulation of HRI, PERK, and ATF4, which is the HRI‐ and PERK/eIF2α/ATF4 signaling axis of stress response and related to skeletal muscle protein synthesis.

### 3.4 Streptomycin induces mitochondrial network fragmentation

**FIGURE 4:**
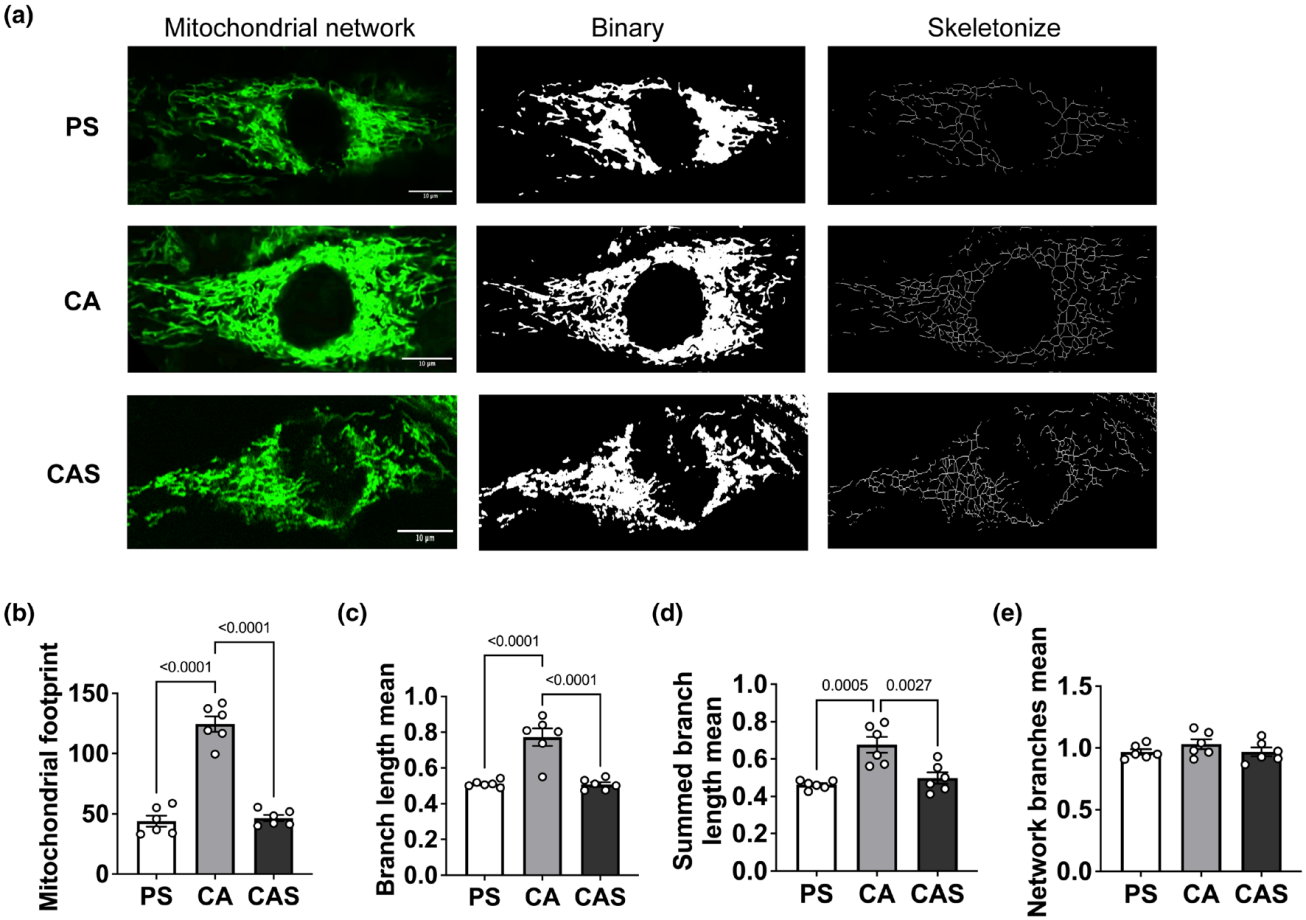
Streptomycin alters mitochondrial networks in C2C12 myotubes. (a) Representative results of MitoTracker staining for mitochondrial labeling of live myotubes in differentiation medium with different antibiotics on day 6. Scale bar: 10 μm. (b) Mitochondrial footprint (the area or volume of the image consumed by mitochondrial signal). (c) Mean branch length (the mean length of all the lines used to represent the mitochondrial structures). (d) Summed mean branch lengths (the mean of the sum of the lengths of branches for each independent structure). (e) Mean network branches (the mean number of attached lines used to represent each structure). Values are expressed as mean ± SD (*n* = 6). PS: Penicillin+streptomycin. CA: Carbenicillin+ampicillin. CAS: Carbenicillin + ampicillin + streptomycin.

To further investigate the impact of streptomycin on mitochondrial structure and function, we visualized mitochondrial networks in C2C12 cells using live‐cell imaging. Representative confocal images are presented in Figure 4a. Cells exposed to streptomycin exhibited a visibly reduced mitochondrial network. We characterized mitochondrial morphology in detail using four parameters and observed that mitochondrial footprint (−64%) (Figure 4b), mean branch length (−34%) (Figure 4c) and summed branch lengths (−30%; Figure 4d), but not mean network branches (Figure 4e), were significantly decreased after exposure to streptomycin.

### 3.5 Streptomycin does not affect mitochondrial respiration, but reduces mitochondrial protein content

**FIGURE 5:**
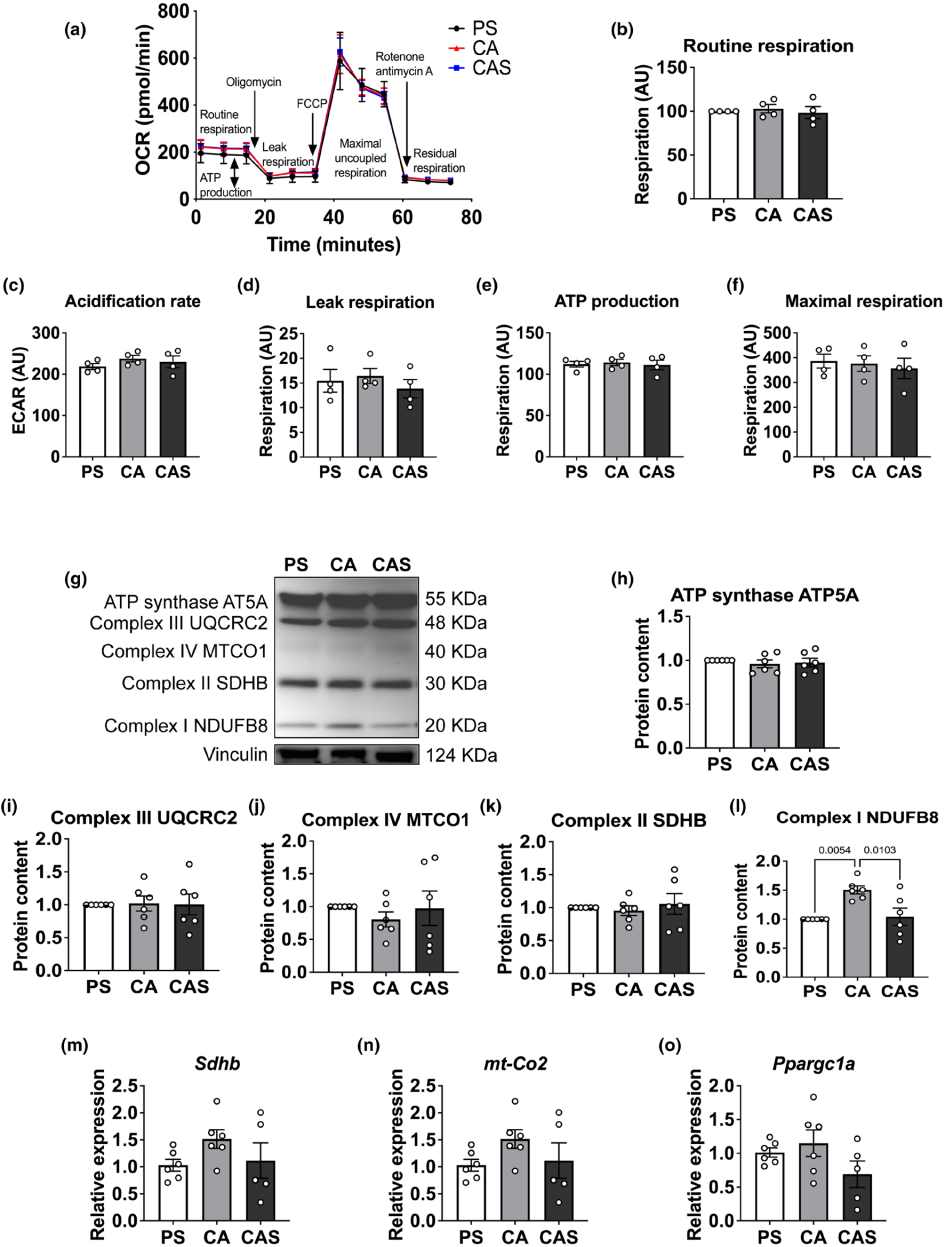
Expression of subunits of mitochondrial complexes in myotubes after exposure to different antibiotics. (a) A typical example of a mitochondrial respiration assay. (b‐f) Baseline (routine) oxygen consumption rates (OCR), Extracellular acidification rates (ECAR), Leak respiration, the oxygen utilization rate for ATP production (routine minus leak respiration), and Maximal uncoupled mitochondrial respiration remained unchanged across the different culture media. Values are expressed as mean ± SD (*n* = 4). (g) Representative results of western immunoblotting using an OXPHOS antibody cocktail in myotubes after 6 days in a differentiation medium with exposure to different antibiotics. Vinculin was used as a loading control. (h–k) No group differences were observed in the protein contents of subunit α of complex V (ATP5A; 55 kDa), cytochrome b‐c1 complex subunit 2 of complex III (UQCRC2l; 48 kDa), mitochondrially encoded cytochrome c oxidase I subunit of complex IV (MTCO1; 40 kDa), and succinate dehydrogenase subunit B (SDHB; 30 kDa). Values are expressed as mean ± SD (*n* = 4–6). (l) NADH dehydrogenase [ubiquinone] 1 β subcomplex subunit 8 of complex I (NDUFB8; 20 kDa), respectively) was significantly lower in conditions where streptomycin was present. Values are expressed as mean ± SD (*n* = 4–6). (m–o) Expression levels of genes related to cellular metabolism (*Sdhb, mt‐Co2, and Ppargc1a*) were not different between groups exposed to different differentiation mediums for 6 days. 18S rRNA was used as internal control for normalization. Values are expressed as mean ± SD (*n* = 5–6). PS: Penicillin+streptomycin. CA: Carbenicillin+ampicillin. CAS: Carbenicillin + ampicillin + streptomycin.

To follow up on the finding of an impaired mitochondrial network upon streptomycin, we next studied mitochondrial respiration. We assessed routine oxygen consumption rates, extracellular acidification rates, leak respiration, the oxygen utilization rate for ATP production, and maximal uncoupled mitochondrial respiration in myotubes (Figure 5b–f). No significant differences were observed in any respiratory parameters across antibiotic conditions.

Lastly, we determined key proteins related to mitochondrial function to study possible mito‐nuclear protein imbalances upon streptomycin exposure. Changes in mitochondrial complexes were detected using a Total OXPHOS Rodent WB Antibody Cocktail. The protein content of NADH dehydrogenase [ubiquinone] 1 β subcomplex subunit 8 of complex I was significantly lower (−33%) after exposure to streptomycin (Figure 5l), but the protein contents of subunits of other mitochondrial complexes were not affected by streptomycin exposure. Although measuring mRNA expression levels of *Sdh*, *mt‐CO2*, and *Ppargc1a*, vital markers for metabolic function, we found no significant differences after exposure to streptomycin; we still can see a relatively clear trend of streptomycin affecting mitochondrial function (Figure 5m–o). Overall, we found that streptomycin has a tendency to affect C2C12 cellular mitochondrial function.

## 4 DISCUSSION

In this study, we report that streptomycin reduces cell growth in C2C12 myoblasts and the protein synthesis rate in cultured C2C12 myotubes. Streptomycin suppressed myotube differentiation but did not affect myoblast proliferation rates. Treating C2C12 myotubes with streptomycin reduced overall protein synthesis rates and subtly altered the translation of mitochondrial‐related proteins and disrupted mitochondrial networks. These results have implications for the use of streptomycin in general cell culture media, particularly when studying muscle hypertrophy, differentiation, and mitochondrial metabolism.

### 4.1 Streptomycin inhibits C2C12 differentiation

Amongst the most widespread antibiotics used for cell culture to prevent bacterial contamination are a combination of penicillin and streptomycin (Ryu et al., 2017). Penicillin antibiotics target the bacterial cell wall and are generally regarded as harmless to eukaryotic cells (Mahmood et al., 2017; Plotz & Davis, 1962). We found cell proliferation not to be affected by streptomycin, similar to previously observed results in cancer cells (Relier et al., 2016). Despite a significant inhibition of protein synthesis in rat hepatocytes, cellular structural integrity was reported to remain intact upon penicillin–streptomycin exposure (Schwarze & Seglen, 1981). However, here we observed that streptomycin inhibits myotube differentiation. Streptomycin reduces C2C12 myoblast differentiation and lowers the fusion index. Our results are in accordance with the observation that the combined penicillin and streptomycin may reduce regenerative potential, growth rate, and differentiation in embryonic stem cells (Cohen et al., 2006). Some studies have found streptomycin to inhibit myogenic differentiation by suppressing the expression of myogenic regulatory factors MyHCf in a dose‐dependent manner (Wedhas et al., 2005). The inhibitory effect of streptomycin on cell differentiation has also been observed in other studies and in other cell lines, such as adipocytes (Llobet et al., 2015). Others have observed that penicillin–streptomycin can alter gene expression and regulation (Ryu et al., 2017) and inhibit the sphere‐forming ability of six cancer cell lines (Relier et al., 2016).

### 4.2 Streptomycin impacts hypertrophy and protein synthesis

To explore the molecular mechanisms underlying the inhibitory effect of streptomycin on C2C12 differentiation, we examined myotube size and protein synthesis rates. In line with results from previous work, where streptomycin significantly decreased force generation of engineered muscle cells after 2 weeks of cell differentiation (Khodabukus & Baar, 2015), we observed changes in the expression of genes coding for proteins of the contractile apparatus, i.e., the decrease in *Myh3* and *Acta1* gene expression. Since contractile force is proportional to fiber diameter (Krivickas et al., 2011), the effect of streptomycin to reduce muscle contraction‐related gene expression is consistent with our observation that streptomycin greatly reduces myotube diameter.

Our puromycin incorporation assay confirmed earlier observations that streptomycin reduced global protein synthesis (Schwarze & Seglen, 1981). The p‐p70S6K/p70S6K ratio, however, was not different after streptomycin exposure in our study, suggesting protein synthesis to be impaired by other mechanisms. A large number of studies have pointed out that most aminoglycoside antibiotics (such as neomycin and paromomycin) contain a central 2‐deoxystreptamine ring (Feldman et al., 2010). In addition to acting on bacterial ribosomes to kill bacteria, they can also interact with the 80S ribosomes in the cytoplasm of eukaryotic organisms, promote the read‐through of premature termination codons in mRNA, reducing translation and protein synthesis (Prokhorova et al., 2017). However, while streptomycin also belongs to the aminoglycoside antibiotics, it does not have such a ring structure, and there is currently no evidence that streptomycin can affect the translation process of cytoplasmic ribosomes. Due to the endosymbiotic “bacterial” origin of mitochondria, impaired translation of ribosomal proteins in mitochondria is considered the main reason for the side effects of streptomycin in eukaryotic cells (Böttger et al., 2001). So a potential mechanism via which streptomycin could inhibit the rate of protein synthesis is by inducing oxidative mitochondrial stress, and as well as endoplasmic reticulum (ER) stress.

Both of these stress types initiate a common signaling output through eukaryotic translation initiation factor 2 subunit α (eIF2α) kinases with high sequence homology, such as protein kinase RNA‐like ER kinase (PERK) and heme‐regulated inhibitor kinase (HRI), to phosphorylate eIF2α, which is an inhibitor of eukaryotic translation initiation factor 2B (eIF2B) (Acharya et al., 2010)–(Moreno‐Gómez‐Toledano et al., 2021, He et al., 2016). Consequently, the global rate of mRNA translation and protein synthesis is reduced (Hinnebusch, 2011). Moreover, phosphorylation of eIF2α specifically activates the translation of ATF4 (Ebert et al., 2022) and phosphorylated eIF2α is in part also involved in ATF4 transcriptional upregulation (Dey et al., 2010). ATF4 is the primary transcriptional regulator for the cellular stress response (Mielnicki & Pruitt, 1991). Its target genes code for cytoprotective proteins involved in protein folding and assembly, metabolism, nutrient uptake, gene expression, alleviation of oxidative stress, and the regulation of apoptosis (Harding et al., 2003; Rozpedek et al., 2016).

To test whether streptomycin had activated the HRI‐ and PERK/eIF2α/ATF4 signaling axis, known as the integrated stress response (ISR) (Tang et al., 2024), we have quantified mRNA expression of HRI and PERK and show that these levels were significantly increased by streptomycin by 50% and 58%, respectively. Moreover, ATF4 mRNA levels were increased by 58% in streptomycin‐treated myotubes in our study. Some evidence shows that HRI and PERK are related to muscle atrophy and ATF4 promotes muscle atrophy (Adams et al., 2017; Ebert et al., 2022). In young adult (2‐month‐old) mice overexpressing ATF4, ATF4 significantly reduced puromycin incorporation into total skeletal muscle protein, suggesting reduced global protein synthesis (Ebert et al., 2015). Together, these data indicate that streptomycin exposure elicits stress responses in myotubes which attenuate the global rate of protein synthesis as well as promote the integrated stress response, potentially involving other pathways of the unfolded protein response (i.e. the IRE1a/XBP1s and ATF6) that affect protein turnover in skeletal muscle (Gallot & Bohnert, 2021).

### 4.3 Streptomycin impacts mitochondrial function

As a potential target of streptomycin‐induced myotubular atrophy and an important target of the stress response, mitochondrial function is of interest. Muscle hypertrophy and atrophy are caused by changes in protein synthesis and degradation, which are highly dependent on mitochondrial energy (Sandri, 2008). In the present study, we found that streptomycin treatment resulted in a lower mitochondrial network integrity. Maintaining the integrity of the mitochondrial network is essential for mitochondrial energy production, because the mitochondria are dynamic organelles that balance fusion and fission processes to meet cellular energy requirements (Adebayo et al., 2021). During mitochondrial fusions and fissions, a dynamic network of mitochondria is formed that tightly regulates mitochondrial shape, size, and number, which distributes energy within the cell more efficiently (Youle & van der Bliek, 2012). It has also been demonstrated that dysregulation of mitochondrial networks and diminished mitochondrial function contribute to sarcopenia (Hood et al., 2019).

A considerable part of the harmfulness linked with aminoglycosides, including streptomycin, could stem from their potential to impair mitochondrial protein synthesis, jeopardizing mitochondrial integrity (Henley 3rd & Schacht, 1988; Hong et al., 2015). Streptomycin was shown to cause blurring, fusion, and cavitation of mitochondrial cristae (Feeney, 2015) along with modifications in mitochondrial structure in retinal ganglion cells (Kogachi et al., 2019). Other widely used antibiotics, such as erythromycin and clindamycin, were similarly reported to inhibit mitophagy and the turnover of the mitochondrial network, whilst showing compromised activity of the mitochondrial respiratory chain (Prajapati et al., 2019).

The respiratory system's largest enzyme is complex I, and several studies have indicated that complex I dysfunction can impact mitochondrial morphology and function in cardiomyocytes, neurons, fibroblasts, and other cells (Harding et al., 2003; Rozpedek et al., 2016; Tang et al., 2024, Wüst et al., 2016). The protein expression of the key subunit of mitochondrial complex I NDUFB8 was decreased upon streptomycin exposure. This mitochondrial complex I subunit is located in the inner‐membrane of the mitochondria and is nuclear‐encoded and assembled in cytoplasmic ribosomes. This implies that some mitochondrial function may be impaired by streptomycin. However, in our study, maximal uncoupled mitochondrial respiration was not different after streptomycin exposure, despite a decrease in complex I protein content upon exposure to streptomycin. Kanamycin (25 μg/mL) decreased mitochondrial basal respiration and maximal respiratory capacity (Kalghatgi et al., 2013). Other antibiotics, such as tetracyclines, similarly showed impairments in energy metabolism through an interruption in mitochondrial proteostasis and physiology within roundworms, fruit flies, and mice to human cell lines (Wang et al., 2015). Doxycycline also can cause a disruption in mito‐nuclear DNA‐derived protein homeostasis in doxycycline‐treated cardiac cells (Wüst et al., 2021). It was also found that culturing engineered muscle in the absence or presence of 100 μg/mL of streptomycin did not affect metabolic protein expression, including glycolytic metabolism related protein (PFK and GLUT4) and mitochondrial enzymes (SDH, Cyt C and ATPsynthase), although streptomycin significantly reduced force production (Khodabukus & Baar, 2015).

A potential explanation for the insignificant changes in metabolism‐related proteins and mitochondrial respiratory function in our study might be that the streptomycin dose was not high enough, as the mitochondrial effects of doxycycline are not conserved under different dosing (Moullan et al., 2015). However, in the present study, we only consider the dose of 100 μg/mL of streptomycin that is commonly and practically used in cell culture. A relatively insensitive respiratory assay may also contribute to the absence of group differences. Another possible explanation is ATF4‐mediated metabolic reprogramming. The above‐mentioned ATF4 may regulate the extent of mitochondrial quality control (MQC) processes and the overall function of the mitochondrial pool (Hood et al., 2019). When mitochondrial stress is initiated, ATF4 upregulates the expression of mitochondrial chaperones, proteases, and enzymes that are required for proper mitochondrial functioning and cycling (Quirós et al., 2017; Zhao et al., 2002). Studies show that myotubes with ATF4 overexpression exhibited higher mitochondrial oxygen consumption rates (OCRs); knocking down ATF4 instead resulted in lower ATP production, as well as basal and maximal respiration, and total OCR when normalized to mitochondrial content, suggesting impaired organelle function (Memme et al., 2023).

## 5 PRACTICAL IMPLICATIONS AND FUTURE WORK

That streptomycin, and potentially other antibiotics of the same class, induces cell stress, reduces muscle differentiation, impairs mitochondrial networks, decreases overall protein synthesis rates, and leads to atrophy, likely has consequences for our understanding of cell culture experiments, but also muscle fiber regeneration and adaptation in vivo. Future experiments with intact muscle can provide in vivo information on the effects of streptomycin on muscle growth, protein synthesis rate, and mitochondrial structure and function, similar to doxycycline (Wüst et al., 2021). Skeletal muscle regeneration is required after microdamage during daily life activities (Arnold et al., 2007). To effectively regenerate skeletal muscle, satellite cells need to be activated and proliferating myoblasts need to be stimulated to fuse into multinucleated myotubes (Ciciliot & Schiaffino, 2010). Due to the inhibitory effect of streptomycin on cell differentiation, people who take streptomycin as medication may experience delayed muscle repair and regeneration, similar to the use of glucocorticoid (dexamethasone) or statins, shown to impair the repair phases of post‐injury skeletal myofibre regeneration (Otrocka‐Domagała et al., 2019). More research is needed to evaluate the effect of streptomycin on the skeletal muscle tissue repair upon injury.

For cell culture experiments, the addition of streptomycin to culture medium alters cell growth and impacts metabolism. As such, this could complicate the interpretation of cell culture studies, as compound interventions can be interpreted as a second‐hit model (Wüst et al., 2020). Because of the role that energy metabolism plays in cell physiology, streptomycin‐induced cell stress may affect the normal physiological function of the entire cell. This means that these add‐on effects may lead to additional cellular adjustments.

However, some study limitations should be acknowledged. A dose–response streptomycin‐only condition would enhance the understanding of streptomycin on C2C12 differentiation and protein synthesis. This requires an antibiotic‐free medium as an appropriate control condition, but any minor bacterial growth would affect myotube growth and mitochondrial structure (Eggelbusch et al., 2022).

## 6 CONCLUSION

The most important finding from this study is that streptomycin inhibited cell differentiation and growth in muscle myotubes. Further, we observed that streptomycin induced oxidative mitochondrial stress and ER stress, reduced protein synthesis rates, altered the mitochondrial network, and impacted the expression of energy metabolism‐related proteins in C2C12 myotubes. These results indicate that streptomycin may induce muscle atrophy by affecting protein synthesis and energy metabolism in skeletal muscle. This study emphasizes the need for caution when interpreting muscle physiology studies that use streptomycin in cell culture, as its effects on cellular processes may confound experimental outcomes. These findings contribute to a broader understanding of the potential negative effects of commonly used antibiotics on muscle health and recovery and raise concerns regarding the use of streptomycin in cell culture experiments.

## FUNDING INFORMATION

This research was funded by the grant from the China Scholarship Council (CSC grant number 202207720075).

## ETHICS STATEMENT

The authors declare that there are no conflicts of interest regarding the publication of this paper.

## Data Availability

The datasets generated and analyzed during the current study are available from the corresponding author upon reasonable request.
